# Care-Related and Maternal Risk Factors Associated with the Antenatal Nondetection of Intrauterine Growth Restriction: A Case-Control Study from Bremen, Germany

**DOI:** 10.1155/2017/1746146

**Published:** 2017-04-04

**Authors:** Sinja Alexandra Ernst, Tilman Brand, Anna Reeske, Jacob Spallek, Knud Petersen, Hajo Zeeb

**Affiliations:** ^1^Leibniz-Institute for Prevention Research and Epidemiology-BIPS, Bremen, Germany; ^2^Federal Institute for Occupational Safety and Health (BAuA), Dortmund, Germany; ^3^Brandenburg University of Technology Cottbus-Senftenberg, Senftenberg, Germany; ^4^Klinik für Frauenheilkunde und Geburtshilfe, Klinikum Links der Weser, Bremen, Germany; ^5^Health Sciences Bremen, University of Bremen, Bremen, Germany

## Abstract

*Objective*. To identify care-related and maternal risk factors for the antenatal nondetection of IUGR.* Methods*. In this hospital-based case-control study we compared antenatally undetected IUGR neonates (cases) to detected IUGR neonates (controls). Data were collected using newborn documentation sheets and standardized personal interviews with the mothers. We calculated antenatal detection rates and used uni- and multivariable logistic regression models to assess the association of antenatal nondetection of IUGR and maternal and care-related factors.* Results*. A total of 161 neonates from three hospitals were included in the study. Suboptimal fetal growth was identified antenatally in *n* = 77 pregnancies while in *n* = 84 it was not detected antenatally (antenatal detection rate: 47.8%). Severity of IUGR, maternal complications, and a Doppler examination during the course of pregnancy were associated with IUGR detection. We did not find statistically significant differences regarding parental socioeconomic status and maternal migration background.* Conclusions*. In our study, about half of all pregnancies affected by suboptimal growth remained undetected. Future in-depth studies with larger study populations should further examine factors that could increase antenatal detection rates for IUGR.

## 1. Introduction

Intrauterine growth restriction (IUGR) can be described as the inability of a fetus to reach its designated growth potential at any gestational age; pregnancies with IUGR are affected by conditions that restrict the normal growth of the fetus [1]. The term IUGR is often used synonymously with small for gestational age (SGA), defined as a birthweight (BW) or estimated fetal weight (EFW) < 10th percentile for gestational age and sex. Fetuses identified as growth restricted, however, comprise a heterogeneous group regarding causal factors, management, and prognosis [2, 3]. Many fetuses or infants with an EFW/BW < 10th percentile are perfectly normal and simply “constitutionally” small [1]. The American College of Obstetricians and Gynecologists Committee highlights that the distinction between normal and pathological growth in clinical practice is challenging [4].

Approximately 3% to 8% of all infants born in developed countries have been identified as growth restricted [5–8]. IUGR is a prenatal condition and is associated with a higher risk for perinatal morbidity and mortality, with risk increasing with severity of the restriction [1]. A recent population-based study confirmed that IUGR is the single largest risk factor for stillbirth, increasing the stillbirth rate fourfold compared to pregnancies with normally grown fetuses; antenatal nondetection further increases the rate by a factor of two [9]. An early antenatal detection, choosing the optimal time and method of delivery, and treatment where appropriate could minimize the risks significantly [9–11]. Umbilical artery Doppler examination is the most valuable tool regarding the prediction of perinatal outcome in growth-restricted fetuses [1] and is accepted as the primary assessment tool regarding diagnosis of IUGR [10, 12]. However, low antenatal detection rates of suboptimal fetal growth through routine fetal ultrasonography have been reported [13, 14]. In fact IUGR has been reported to be antenatally detected only in one-third (25% to 32%) of pregnancies with suboptimal fetal growth [15, 16].

Apart from the difficulty to distinguish between healthy SGA fetuses and pathological IUGR cases, reasons for the antenatal nondetection of IUGR have not been well elucidated yet. IUGR is a heterogeneous condition with various underlying maternal, placental, or environmental causes. Antenatal care use and maternal characteristics such as socioeconomic status (SES) and migration background may also play a role [17–19].

The aim of this study was to identify care-related and maternal risk factors for the antenatal nondetection of IUGR and to investigate if there are specific groups with a higher chance of nondetected suboptimal fetal growth.

## 2. Material and Methods

### 2.1. Study Design

This hospital-based case-control study was carried out in cooperation with three obstetric units in the federal city-state of Bremen, Germany. Our study design differed from the traditional case-control study design, as we did not compare IUGR cases to healthy controls; instead we compared antenatally undetected IUGR neonates (cases) to detected IUGR neonates (controls). A detailed description of the study design and methods is described in the study protocol [20]. The study region covered a geographical area of some 420 km^2^ with 669,915 residents and 6,397 deliveries in 2012. 4,935 of these deliveries took place in the three cooperating hospitals, out of a total of five hospitals with an obstetric unit in the study region. In Germany antenatal and perinatal care is covered by the health insurance system. Health insurance is compulsory and provided by the statutory health insurances (roughly 90% of the German population) or by private health insurances [21].

### 2.2. Recruitment of Participants

From January 2013 to June 2015 mothers and their newborns with a birthweight <10th percentile in relation to gestational age and sex (SGA) were eligible for the study and invited for participation. Obstetricians and study nurses classified newborns as SGA based on the routinely used population-based percentile values for newborns in Germany by Voigt et al. [22, 23]. Mothers were initially informed about the study by the attending obstetrician during their hospitalization or if they had already left the hospital via postal mail by the study nurses. Study nurses sent reminders three and six weeks after the initial contact, including a nonresponder questionnaire after six weeks. Study materials (i.e., study information, study flyer) were translated into Russian and Turkish language. Where required, the maternal interviews were conducted in one of these languages. The whole study procedure was pretested among a small sample of mothers prior to the recruitment phase.

### 2.3. Data Collection

We designed a newborn documentation sheet to record basic information, such as birthweight, birth length, head circumference, Apgar score, umbilical cord blood pH, gestational age at birth, complications at birth, and mode of delivery. The basic information was documented by obstetricians at the time of birth or by study nurses based on the birth records. Details on the IUGR diagnosis, such as timing (i.e., antenatal versus at birth) were added by the attending obstetrician or pediatrician in the hospital. For all mothers who declined to participate in the study, the newborn documentation sheet was also filled out by the attending obstetricians or by study nurses (basic information only; no information on IUGR diagnosis).

All mothers who consented to the study were interviewed at home after they were discharged from the hospital. The questionnaire was developed in close cooperation with obstetricians and designed as a standardized CAPI/CATI of approximately 45 mins duration. Aspects covered by the questionnaire were sociodemographic information, medical conditions, and complications/diseases during pregnancy, for example, maternal vascular diseases, infections during pregnancy, preeclampsia, placental anomalies, anomalies of the uterus, risk factors for IUGR during pregnancy such as smoking, alcohol consumption, and illicit drug use, maternal height and weight to determine the maternal prepregnancy BMI, maternal weight gain during pregnancy, and parity and number of pregnancies, as well as use, timing, and content of antenatal care. The interviews were conducted by trained project personnel and medical conditions were confirmed by data of the pregnancy record books.

### 2.4. Case-Control Definition

Cases were defined as neonates with an IUGR not detected antenatally; that is, the IUGR diagnosis was initially established at the time of birth or during the first medical check-up after birth (newborn documentation sheet) and the mother did not report any IUGR diagnosis in the personal interview.

Controls were defined as neonates whose IUGR was positively identified antenatally; that is, the diagnosis including date either was documented in the newborn documentation sheet or was stated by the mother in the personal interview. Newborns with a suspected (but not confirmed) IUGR diagnosis documented in the newborn documentation sheet were also defined as controls.

### 2.5. Variables

Maternal migration background was defined as being born in a foreign country and/or having a nationality other than German. Information on household income, education, and occupation were combined into a composite socioeconomic status measure (SES; low, middle, high) as proposed by Winkler and Stolzenberg [24].

Gestational weight gain was calculated based on prepregnancy body-mass-index (BMI) for underweight, normal weight, overweight, and obese women as recommended by* The Institute of Medicine (IOM)* [25] and then categorized into (1) lower than adequate, (2) adequate, and (3) higher than adequate.

Further maternal factors were age (<35 versus ≥35 yrs), parity, number of pregnancies (gravidity), maternal prepregnancy BMI, coffee intake, tobacco consumption, and illicit drug use during pregnancy and maternal complications/diseases during pregnancy, that is, maternal vascular diseases (e.g., hypertension, preeclampsia), infections during pregnancy (e.g., toxoplasmosis), and malformation of the uterus and placental anomalies (e.g., placenta praevia).

The severity of IUGR diagnosis was determined using different cut-off limits for the BW percentile, that is, BW percentile <3rd, ≥3rd–<5th, and ≥5th–<10th, for gestational age and sex. Fetal sex, multiple gestation, and fetal anomalies, for example, trisomy 13, trisomy 18, trisomy 21, and congenital malformations, were included. Further outcome parameters were 1-minute and 5-minute Apgar score (normal: 7–10; minor depression: 4–6; severe depression: 0–3) and umbilical cord blood pH (ideal: >7.3; normal: 7.2–7.29; minor acidification: 7.1–7.19; moderate acidification: 7.0–7.09; severe acidification: <7.0).

We constructed an index to assess adequate antenatal care use [26, 27], by combining gestational age of the first antenatal care visit and the total number of visits, taking the gestational age at birth into account. We used the recommended schedule of the maternity guidelines for Germany as the basis of our index. The values range from 1 to 4 and the index was constructed separately for nulliparae and multiparae. For nulliparae, our index has the following 4 categories of antenatal care use in case of a full-term pregnancy: (1) adequate use—a minimum number of 8 visits and a first visit before gestational age of 12 weeks; (2) less adequate use—less than 8 visits and a first visit before gestational age of 12 weeks; (3) inadequate use—a minimum number of 8 visits and a first visit at gestational age after 12 weeks; (4) more inadequate use—less than 8 visits and a first visit at gestational age after 12 weeks. For the multiparae we used basically the same categories, except that the minimum total visits for a full-term pregnancy were 6 visits. Further care-related factors included the number of routine ultrasonography and Doppler examinations during pregnancy, any hospitalization during pregnancy, number of admissions of newborns to neonatal care unit (NCU), and mode of delivery.

### 2.6. Statistical Analysis

The incidence of SGA was calculated for the three participating hospitals by dividing all births <10th percentile for gestational age and sex by all recorded births in this period. This calculation was based on the recorded basic information of all births in the participating hospitals during the years 2013 and 2014. Antenatal detection rates of IUGR were calculated by dividing all newborns with antenatally identified IUGR by the whole study sample and stratified by different cut-off limits for IUGR identification (i.e., birthweight <10th, <5th, and <3rd percentile for gestational age and sex). We examined associations between care-related and maternal determinants and nondetection of IUGR in univariate and multivariable logistic regression models, using Odds Ratios (OR) with 95% confidence intervals (CI). Multivariable models were adjusted for maternal migration background, socioeconomic status, maternal age (<35 yrs versus ≥35 yrs), birthweight percentile (<3rd, ≥3rd–<5th, and ≥5th–<10th), complications/diseases during pregnancy, Doppler examination, fetal anomalies, and multiple gestation. In sensitivity analyses we examined differences in the applied method of case-control identification and source of information used, that is, newborn documentation sheet and CAPI. Differences in birth-related characteristics between responders and nonresponders were tested using chi-square tests or *t*-tests, where appropriate. The study was planned to detect moderate to large differences in terms of risk factors for nondetection of IUGR (OR > 2.0), with a statistical power of 0.8 and a 95% CI with an estimated sample size of *n* = 260.

### 2.7. Ethics Approval and Consent to Participate

Ethical approval for all study procedures was obtained from the ethics review board of the Bremen Medical Association. All women who delivered an SGA newborn in one of the cooperating hospitals received written and oral information about the study. All participating women had to give written informed consent for data collection.

## 3. Results

The total number of births during the whole 2.5-year recruitment period was 12,926 in the three participating maternity hospitals. A total of *n* = 1,087 (8.4%) newborns had a birthweight <10th percentile for gestational age and sex at the time of birth and were invited for study participation. Fifteen percent of mothers (*n* = 163) participated in the study. We excluded two participants due to a birthweight ≥10th percentile for gestational age and sex and no documented IUGR diagnosis in newborn documentation sheet or maternal survey data. A comparison of neonates' birth characteristics and outcomes between participants and nonparticipants (*n* = 926; basic information of newborn documentation sheet) showed a statistically significant lower birthweight on average for participants as compared to nonparticipants (mean birthweight (gram) 2477.4 ± 544.9 versus 2579.7 ± 432.5; *p* value: 0.025) (Additional File 1 in Supplementary Material available online at https://doi.org/10.1155/2017/1746146). In total, *n* = 51/926 women who declined to participate in the study filled out the nonresponder questionnaire. The main reasons for nonparticipation were lack of time, language barriers, and no interest in scientific studies in general.

As outlined in Table 1, 20.5% (*n* = 33) of participating mothers had a migration background and only a small number of mothers with low SES participated in our study (high: 51.6%; middle: 41.6%; low: 6.8%). The age distribution among cases and controls was similar, as was the distribution by SES. The proportion of mothers with migration background was slightly higher among cases (23.8%; *n* = 20) than controls (19.9%; *n* = 13) (Table 1). Among controls the proportion of more severe suboptimal fetal growth was higher (<10th percentile: 32.5%; <5th percentile: 14.3%; <3rd percentile: 53.2%) than cases (<10th percentile: 57.1%; <5th percentile: 7.1%; <3rd percentile: 35.7%) (Table 2). None of the mothers stated any illicit drug use during pregnancy.

### 3.1. Antenatal Detection of IUGR

Suboptimal fetal growth was identified antenatally in *n* = 77 pregnancies (controls) while in *n* = 84 (cases) it remained undetected (antenatal detection rate: 47.8%). The antenatal detection rate was highest in newborns with a birthweight <5th percentile (64.7%) and lowest in newborns with a birthweight <10th percentile (34.2%) in relation to gestational age and sex. Among newborns with a birthweight <3rd percentile the antenatal detection rate was slightly lower (57.7%) as compared to newborns with a birthweight <5th percentile (Table 3).

### 3.2. Factors Associated with Nondetection of IUGR

In adjusted models, we identified three factors (severity of IUGR, presence of maternal complications/diseases during pregnancy, and Doppler examination during the course of pregnancy) that were associated with the antenatal nondetection of IUGR (Table 4). Newborns with a birthweight <10th percentile for gestational age and sex were about three times more likely to remain antenatally undetected as compared to newborns with a birthweight <3rd percentile for gestational age and sex (OR 2.82; 95%-CI [1.31, 6.10]) (Table 4). The odds for antenatal nondetection of IUGR were markedly reduced for mothers who had any complications/diseases during pregnancy (OR 0.38; 95%-CI [0.18, 0.79]). The use of Doppler examination during the course of pregnancy also reduced the odds of antenatal nondetection of IUGR significantly (OR 0.13; 95%-CI [0.04, 0.40]).

We did not find statistically significant associations between antenatal nondetection of IUGR and maternal SES or migration background in univariate as well as multivariable regression models (Table 4). However, in multivariable analyses, the point estimate indicated that antenatal nondetection of IUGR is about two times more likely in women with a migration background (OR 1.8; 95%-CI [0.68, 4.56]) than nonmigrants, although it was not statistically significant.

## 4. Discussion

The aim of this paper was to identify care-related and maternal risk factors for the antenatal nondetection of fetal growth restrictions, specifically IUGR. Overall, 8.0% (*n* = 1,087) of all newborns in the cooperating maternity hospitals during study period were SGA, which is in line with other West European studies [6–8, 28].

In our study suboptimal fetal growth was antenatally identified in less than half of the cases as determined perinatally. As compared to the sensitivities reported in observational studies of the late 1990s and early 2000 (25–32%) [15, 16], our study results indicate that IUGR detection rates did not substantially increase over the last 15 years. In line with our findings, a more recent US study reported similar low antenatal detection rates for IUGR of 25% [29]. However, the detection rates found in our study have to be interpreted cautiously. There is a marked heterogeneity in our study population regarding the severity of IUGR. However, in fact it seemed that the majority of included neonates (54.7%) as compared to nonresponders had more severe growth restrictions (birthweight <5th percentile). Our findings indicate that the antenatal detection rate increases with severity of the growth restriction. However, even among the newborns below the 5th percentile, only approximately half of the cases were identified antenatally, a finding that can be seen as indicating a quality problem in antenatal care.

We identified three factors that influenced IUGR detection. A higher severity of the growth restriction, maternal complications/diseases during pregnancy, and a Doppler examination during the course of pregnancy led to higher antenatal detection rates in our study. Similar to this, findings of a recent US multicenter cohort study including 11,487 births showed that maternal complications, an ultrasonography examination with measurement of EFW within four weeks of birth, gestational age at delivery, and a higher severity of the growth restriction increased antenatal detection rates. Hispanic ethnicity was associated with a higher risk of antenatal nondetection (RR 2.4; 95% CI [1.4, 4.2]) [29]. Both our study and larger other studies thus indicate that clinical alertness towards maternal and fetal morbidity and the use of Doppler ultrasonography are core factors that can reduce undetected fetal growth restrictions and their consequences. However, it is likely that a suspected IUGR was in many cases the reason for the Doppler examination because the latter is the primary method for diagnosing IUGR. Nonetheless the detected association confirmed that the performance of a Doppler examination during the course of pregnancy is of great value for the detection of suboptimal fetal growth.

Previous studies have reported differences in use and timing of antenatal care between pregnant women depending on SES and migration background which may lead to an increased risk for adverse pregnancy and birth outcomes for the social disadvantaged [17–19, 30]. Our results showed no statistically significant associations between these variables and antenatal IUGR detection. However, the point estimate indicated twofold increased odds for nondetection of suboptimal fetal growth among women with a migration background. This finding calls for further research with larger study populations and a more differentiated operationalization of migration background. The missing social gradient in the detection rates may be due to the small number of study participants with a low SES.

### 4.1. Strengths and Limitations

This is one of so far very few studies from Germany explicitly investigating care-related as well as maternal risk factors influencing the (non)detection of suboptimal fetal growth. The interviews were pretested with mothers of neonates who were diagnosed with SGA or IUGR to ensure clarity and feasibility of the interview questions, language, structure, and time needed. A further strength is the relatively high proportion of mothers with migration background included in the study. The main limitation of this study is the low response rate. The recruitment of cases and controls in this study was a particular challenge. Firstly, the incidence of suboptimal fetal growth is relatively low. Secondly, it could be assumed that the severity of suboptimal fetal growth influenced the willingness to participate in the study. Mothers whose newborns had severe growth restriction may have declined to participate in the study as they would want to focus their full attention on their infants. However, our comparison of neonates' birth characteristics of participants and nonparticipants showed that newborns of participating mothers had a lower birthweight on average as compared to newborns of mothers who declined to participate (Supplementary Material). Therefore we believe that this type of selection bias is rather unlikely. Furthermore, the percentage of mothers who smoked during pregnancy in our study (10.7%) was similar to the data presented by Kuntz and Lampert on the percentage of mothers who smoked during pregnancy (12.1%), which are based on the German Health Interview and Examination Survey for Children and Adolescents (KiGGS) [31]. All mothers who delivered an SGA newborn in one of the three participating maternity units had an equal chance to participate in the study. However, we cannot fully rule out that there is some extent of selection bias, due to unique characteristics of the mothers who agreed to participate in the study, as their babies were of significantly lower birthweight as compared to the nonparticipants.

Due to the very limited time for recruitment of individual mothers in the maternity units, the shift work, and extensive work load, obstetricians were not always able to inform the mothers about the study and to invite them to participate. The majority of mothers were exclusively invited to participate by written letters. In an attempt to address these issues we offered three interview options, either a CAPI directly in the hospital, a CAPI after birth at home, or a CATI. Several reminders were sent and study material and the interview were translated in different languages to cater for the main migrant groups living in the study region. Nonetheless, our recruitment aim could not be fully reached such that true associations may have remained undetected in our analyses.

## 5. Conclusions

IUGR detection rates do not seem to have substantially increased since the late 1990s, as about half of the pregnancies affected by suboptimal fetal growth remain undetected under routine conditions. Several clinical and care-related factors reduce the risk that IUGR remains undetected. A migration background of the mother may increase nondetection odds, but further studies with larger sample sizes are warranted. A direction for future research could be to examine whether a mandatory Doppler examination (at least for some subgroups) increases antenatal detection rates for IUGR. Our study data can feed into ongoing international efforts to investigate antenatal care explicitly addressing IUGR detection and diagnosis [32].

## Supplementary Material

Comparison of neonates' birth characteristics and outcomes between participants and nonparticipants based on basic information of the newborn documentation sheet.

## Figures and Tables

**Table 1:** Maternal characteristics in total and stratified by cases and controls.

Maternal characteristics	Total (*n* = 161) % (*n*)	IUGR detected (controls) (*n* = 77) % (*n*)	IUGR undetected (cases) (*n* = 84) % (*n*)
Age			
<35 yrs	65.8 (106)	68.8 (53)	63.1 (53)
≥35 yrs	34.2 (55)	31.2 (24)	36.9 (31)
Maternal migration background			
Yes	20.5 (33)	19.9 (13)	23.8 (20)
No	78.3 (126)	81.8 (63)	75.0 (63)
Missing value	1.2 (2)	1.3 (1)	1.2 (1)
Socioeconomic status			
High	51.6 (83)	49.4 (38)	53.6 (45)
Middle	41.6 (67)	44.2 (34)	39.3 (33)
Low	6.8 (11)	6.5 (5)	7.1 (6)
Prepregnancy BMI (kg/m^2^)			
Normal	66.5 (107)	62.3 (48)	70.3 (59)
Overweight	18.0 (29)	18.2 (14)	17.9 (15)
Obese	15.5 (25)	19.5 (15)	11.9 (10)
Weight gain during pregnancy			
Higher than adequate	33.5 (54)	37.7 (29)	29.8 (25)
Adequate	32.9 (53)	31.2 (24)	34.5 (29)
Lower than adequate	33.5 (54)	31.2 (24)	35.7 (30)
Parity^*∗*^			
Nulliparous	55.9 (90)	58.4 (45)	53.6 (45)
Multiparous	44.1 (71)	41.6 (32)	46.4 (39)
Number of pregnancies^*∗*^			
≤1	79.5 (128)	76.6 (59)	82.2 (69)
2–≤3	17.4 (28)	19.5 (15)	15.4 (13)
≥4	3.1 (5)	3.9 (3)	2.4 (2)
Complications/diseases during pregnancy			
Yes	44.7 (72)	54.5 (42)	35.7 (30)
No	55.3 (89)	45.5 (35)	64.3 (54)
Ultrasound examinations			
<3	0.6 (1)	1.3 (1)	0 (0)
3–8	58.4 (94)	50.6 (39)	65.5 (55)
≥9	41.0 (66)	48.1 (37)	34.5 (29)
Doppler examination			
Yes	80.1 (129)	92.2 (71)	69.0 (58)
No	19.9 (32)	7.8 (6)	31.0 (26)
Index antenatal care use			
Adequate	93.2 (150)	92.2 (71)	94.0 (79)
Less adequate or inadequate	6.8 (11)	7.8 (6)	6.0 (5)
Hospitalization during pregnancy			
Yes	22.4 (36)	27.3 (21)	17.9 (15)
No	77.6 (125)	72.7 (56)	82.1 (69)
Alcohol consumption during pregnancy			
Yes	2.5 (4)	1.3 (1)	3.6 (3)
No	96.9 (156)	98.7 (76)	95.2 (80)
Missing value	0.6 (1)	0 (0)	1.2 (1)
Tobacco consumption during pregnancy			
Yes	10.6 (17)	10.4 (8)	10.7 (9)
No	88.8 (143)	89.6 (69)	88.1 (74)
Missing value	0.6 (1)	0 (0)	1.2 (1)

^*∗*^Excluding current pregnancy/birth.

**Table 2:** Newborn characteristics in total and stratified by cases and controls.

Newborn characteristics	Total (*n* = 161) % (*n*)	IUGR detected (controls) (*n* = 77) % (*n*)	IUGR undetected (cases) (*n* = 84) % (*n*)
Sex			
Male	50.9 (82)	50.6 (39)	51.2 (43)
Female	49.1 (79)	49.4 (38)	48.8 (41)
Multiple gestation			
Yes	9.3 (15)	11.7 (9)	7.1 (6)
No	90.7 (146)	88.3 (68)	92.9 (78)
Birthweight percentile			
<10th percentile^1^	45.3 (73)	32.5 (25)	57.1 (48)
<5th percentile	10.6 (17)	14.3 (11)	7.1 (6)
<3rd percentile	44.1 (71)	53.2 (41)	35.7 (30)
Fetal anomalies			
Yes	8.1 (13)	11.7 (9)	4.8 (4)
No	95.0 (153)	88.3 (68)	95.2 (80)
Number of admissions to NCU^2^			
Yes	23.6 (38)	35.1 (27)	13.1 (11)
No	76.4 (123)	64.9 (50)	86.9 (73)
Apgar score			
1 min			
Severe depression	1.2 (2)	2.6 (2)	—
Minor depression	6.2 (10)	6.5 (5)	6.0 (5)
Normal	91.9 (148)	90.9 (70)	92.9 (78)
5 min			
Severe depression	—	—	—
Minor depression	2.5 (4)	3.9 (3)	1.2 (1)
Normal	96.9 (156)	96.1 (74)	97.6 (82)
Umbilical cord blood pH			
Ideal	48.4 (78)	48.1 (37)	48.8 (41)
Normal	35.4 (57)	33.8 (26)	36.9 (31)
Minor acidification	11.2 (18)	13.0 (10)	9.5 (8)
Moderate acidification	1.2 (2)	1.3 (1)	1.2 (1)
Missing value	3.7 (6)	3.9 (3)	3.6 (3)
Mode of delivery			
Vaginal	50.3 (81)	41.6 (32)	58.3 (49)
Cesarean section (elective)	13.7 (22)	22.1 (17)	6.0 (5)
Cesarean section (secondary)	25.5 (41)	29.9 (23)	21.4 (18)
Other	10.5 (17)	6.5 (5)	14.3 (12)

^1^Including  *n* = 4 newborns with a birthweight >10th percentile for gestational age and sex, but with (antenatal) diagnosis of IUGR/SGA.

^2^NCU: neonatal care unit.

**Table 3:** Antenatal detection rates according to birthweight percentiles (<3rd, ≥3rd–<5th, ≥5th–<10th percentile for gestational age and sex).

*n* = 161	Participating mothers % (*n*)
Antenatal detection rate	47.8% (77/161)
≥5th–<10th birthweight percentile	34.2% (25/73)
≥3rd–<5th birthweight percentile	64.7% (11/17)
<3rd birthweight percentile	57.7% (41/71)

^*∗*^Classification based on combined evaluation of both information sources (newborn documentation sheets, personal interview).

**Table 4:** Association between antenatal nondetection of IUGR and maternal and care-related factors; univariate and multivariable regression analyses.

*n* = 161	Univariate regression model Odds Ratio [95%-CI]	Multivariable regression model^1^ Odds Ratio [95%-CI]
Maternal migration background		
Yes	1.54 [0.71, 3.36]	1.76 [0.68, 4.56]
No	Reference	Reference
Socioeconomic status		
High	Reference	Reference
Middle	0.82 [0.43, 1.56]	0.75 [0.35, 1.61]
Low	1.01 [0.29, 3.58]	0.47 [0.10, 2.27]
Maternal age		
<35 years	0.77 [0.40, 1.49]	0.94 [0.44, 2.02]
≥35 years	Reference	Reference
Birthweight percentile		
<10th percentile	**2.62 [1.34, 5.15]**	**2.82 [1.31, 6.10]**
<5th percentile	0.75 [0.25, 2.24]	0.73 [0.22, 2.49]
<3rd percentile	Reference	Reference
Complications/diseases during pregnancy		
Yes	**0.46 [0.25, 0.87]**	**0.38 [0.18, 0.79]**
No	Reference	Reference
Doppler examination		
Yes	**0.19 [0.07, 0.49]**	**0.13 [0.04, 0.40]**
No	Reference	Reference
Fetal anomalies		
Yes	0.38 [0.11, 1.28]	0.21 [0.05, 0.97]
No	Reference	Reference
Multiple gestation		
Yes	0.58 [0.20, 1.72]	0.45 [0.12, 1.61]
No	Reference	Reference
BMI (kg/m^2^)		
Normal	Reference	—
Overweight	0.87 [0.38, 1.98]	
Obese	0.54 [0.22, 1.32]	
Weight gain during pregnancy		
Higher than adequate	0.71 [0.0.33, 1.53]	—
Adequate	Reference	
Lower than adequate	1.03 [0.48, 2.22]	
Tobacco consumption		
Yes	1.05 [0.38, 2.87]	—
No	Reference	
Parity^2^		
Nulliparous	Reference	—
Multiparous	1.22 [0.65, 2.27]	
Index antenatal care use		
Adequate	Reference	—
Less adequate or inadequate	0.75 [0.22, 2.56]	
Hospitalization during pregnancy		
Yes	0.58 [0.27, 1.23]	—
No	Reference	
Newborn sex		
Male	1.02 [0.55, 1.90]	—
Female	Reference	

^1^Adjusted for maternal migration background, socioeconomic status, maternal age (<35 yrs versus ≥35 yrs), birthweight percentile (<10th–≥5th, <5th–≥3rd, <3rd), complications/diseases during pregnancy, Doppler examination, fetal anomalies, and multiple gestation.

^2^Excluding current pregnancy/birth.

*Note*. Numbers of entries given in bold indicate a significant association.
