# Supplementary material for: Care-Related and Maternal Risk Factors Associated with the Antenatal Nondetection of Intrauterine Growth Restriction: A Case-Control Study from Bremen, Germany
Source: Biomed Res Int. 2017 Apr 4;2017:1746146. doi: 10.1155/2017/1746146 (PMC5394345; doi:10.1155/2017/1746146)
Supplement: Supplementary file 1 — Comparison of neonates' birth characteristics and outcomes between participants and nonparticipants based on basic information of the newborn documentation sheet. [file 1746146.f1.docx]

**Additional file 1.** Comparison of neonates’ characteristics, non-responding and responding participants

|  | **Total**  N=1087 | **Participants**  n=161 | **Non-Responder***  n=926 |  |
| --- | --- | --- | --- | --- |
|  | **% (n)** | **% (n)** | **% (n)** | **p-value** |
| **Sex**  Male  Female | 48.2 (520)  51.8 (558) | 50.9 (82)  49.1 (79) | 47.8 (438)  52.2 (479) | 0.458 |
| **Gestational age at delivery (wks)** | | | | |
| Mean (SD) | 38.6 (2.3) | 38.3 (2.8) | 38.6 (2.2) | 0.062 |
| **Apgar (1 min)**  Severe depression  Minor depression  normal | 1.2 (11)  4.3 (38)  94.5 (840) | 1.3 (2)  6.3 (10)  92.5 (148) | 1.2 (9)  3.8 (28)  94.9 (692) | 0.429 |
| **Apgar (5 min)**  Severe depression  Minor depression  normal | -  1.0 (9)  99.0 (880) | -  2.5 (4)  97.5 (156) | -  0.7 (5)  99.3 (724) | 0.061 |
| **umbilical cord blood pH**  ideal  normal  minor acidification  moderate acidification  Mean (SD) | 42.5 (462)  27.8 (302)  8.6 (93)  1.2 (13)  7.29 (0.08) | 50.3 (78)  36.8 (57)  11.6 (18)  1.3 (1)  7.28 (0.79) | 53.6 (384)  34.2 (245)  10.5 (75)  1.7 (12)  7.29 (0.08) | 0.855  0.112 |
| **Mode of delivery**  Vaginal  Cesarean section (elective)  Cesarean section (secondary)  Other | 61.1 (660)  10.9 (118)  20.0 (216)  8.1 (87) | 50.3 (81)  13.7 (22)  25.5 (41)  10.5 (17) | 62.9 (579)  10.4 (96)  19.0 (175)  7.6 (70) | **0.027** |
| **Complications at birth**  Yes  No | 79.6 (854)  20.4 (219) | 81.1 (129)  18.9 (30) | 79.3 (725)  20.7 (189) | 0.601 |
| **Head circumference, cm**  Mean (SD) | 33.1 (1.9) | 32.8 (2.2) | 33.1 (1.8) | 0.057 |
| **Birth lengths, cm**  Mean (SD) | 48.0 (3.2) | 47.5 (4.4) | 48.1 (3.0) | 0.082 |
| **Birthweight**  Mean (SD) | 2564.6 (451.3) | 2477.4 (544.9) | 2579.7 (432.5) | **0.025** |
| **Birthweight percentile**  <10^th^ percentile  <5^th^ percentile  <3^rd^ percentile | 52.1 (566)  10.9 (119)  37.0 (402) | 45.3 (73)  10.6 (17)  44.1 (71) | 53.2 (493)  11.0 (102)  35.7 (331) | 0.119 |

*****with filled out newborn documentation sheet (basic information only)
